# Comparison of a daily and alternate-day photobiomodulation protocol in the prevention of oral mucositis in patients undergoing radiochemotherapy for oral cancer: a triple-blind, controlled clinical trial

**DOI:** 10.4317/medoral.26436

**Published:** 2024-04-14

**Authors:** Flávia Marília Azeredo de Oliveira, Marcela Maria Fontes Borges, Cássia Emanuella Nóbrega Malta, José Fernando Bastos de Moura, Clarissa Pessoa Fernandes Forte, Jennifer Vianna Barbosa, Paulo Goberlânio de Barros Silva, Thinali Sousa Dantas

**Affiliations:** 1 Department of Dentistry, Unichristus, Fortaleza, Ceará, Brazil; 2 Department of Dental Clinic, Division of Oral Pathology, Faculty of Pharmacy, Dentistry and Nursing, Federal University of Ceará, Fortaleza, Ceará, Brazil; 3 Hospital Haroldo Juaçaba, Ceará Cancer Institute, Fortaleza, Ceará, Brazil

## Abstract

**Background:**

Preventive Photobiomodulation Therapy (PBMT) significantly reduces oral mucositis (OM) severity in patients undergoing Radiochemotherapy (RCT) for the treatment of oral cancer, but daily applications generate cost, overload the dental team, and reduce the number of patients assisted.To evaluate the effectiveness of two PBMT protocols in preventing OM in patients undergoing RCT for oral cancer.

**Material and Methods:**

16 patients diagnosed with oral cancer undergoing RCT were included, equally divided into two groups: a group treated daily with PBMT, and another group also submitted to daily treatment, however, performing the application of PBMT every three days, interspersed with a simulation of PBMT (placebo). A red laser was used (~660 nm), 0.1W power, 1J of energy applied per point, 9 points per area (labial mucosa, buccal mucosa, lateral borders of the tongue, body of the tongue, and floor of the mouth) from the beginning of RCT until the end of the oncological treatment. Daily assessments were performed regarding OM scores, the World Health Organization (WHO) pain scale, and the visual analog scale (VAS). Weight, salivary flow (SGAPP), OHIP-14, and DMFT were evaluated on the initial and final days of RT. OM incidence and clinical data were compared by Pearson's chi-square test or Fisher's exact test. Pain and other scale scores were compared using the Mann-Whitney and Friedman/Dunn tests (SPSS v20.0 *p*<0.05).

**Results:**

In the group with PBMT on alternate days, there was an increase in the frequency of grade 2 and grade 3 oral mucositis and an increased risk of grade 2 oral mucositis, in addition to higher mean pain scores and greater reduction in salivary flow.

**Conclusions:**

The daily PBMT protocol proved more effective in controlling the frequency and severity of OM, pain, and salivary flow.

** Key words:**Stomatitis, oral neoplasms, radiotherapy, antineoplastic combined chemotherapy protocols, low-intensity light therapy.

## Introduction

The first line of treatment for oral cancer is surgical resection, especially in the early stages (1). However, in most cases, radiotherapy (RT) is associated with the treatment, either curatively or as an adjuvant to the surgical treatment, especially when the tumor has unfavorable pathological characteristics or resection with compromised margins. Chemotherapy, in these cases, can also be associated with RT to induce and potentiate cell death (2,3).

Antineoplastic treatment, especially RT in the oral cavity, can have a negative impact on patients' quality of life because, as well as damaging the DNA of neoplastic cells, RT also affects adjacent healthy tissues (4,5). This can lead to numerous side effects, such as oral mucositis (OM), which is caused by mitotic death directly induced by radiation in the basal cells of the mucosal epithelium (6).

Mucosal lesions generally occur in the first few days after the end of the chemo/radiotherapy conditioning regimen, peaking between days 7 and 11 (7). This adverse effect has a direct impact on the quality of life during the treatment of these patients and can lead to fever, an increased risk of infection, the need for parenteral nutrition, and more days of opioid medication administration (8). The combination of these factors results in an increase in the length of hospital stay and treatment costs. This increases susceptibility to opportunistic infections, increasing the risk of morbidity and mortality (9).

Low-intensity laser therapy, also known as photobiomodulation therapy (PBMT), is widely tested and has the highest level of evidence for preventing OM. PBMT promotes photochemical changes in target tissues without causing structural loss (10).

Among the biological effects related to PBMT, we can mention the increase in mitochondrial ATP production, the stimulation of lymphocytes, the activation of mast cells, the proliferation of various types of cells, and anti-inflammatory effects (11). Thus, the biostimulatory effect for tissue repair is possible with PBMT, increasing local circulation, cell proliferation, and collagen synthesis (11).

Several studies have shown the effectiveness of daily protocols for preventing OM (12,13). However, daily applications of PBMT during RCT can be costly for the service, overburden the dental team, and reduce the number of patients that could receive the treatment. Therefore, this study aimed to evaluate the effectiveness of two PBMT protocols in preventing OM in patients undergoing RCT for oral tumors.

## Material and Methods

- Study design and ethical considerations

This phase III, randomized, triple-blind, controlled clinical trial registered in the Brazilian Clinical Trials Registry (www.ensaiosclinicos.gov.br) complies with all CONSORT guidelines for clinical trials.

This study was submitted to the Scientific Technical Committee of Faculdade Rodolfo Teófilo/Instituto do Câncer do Ceará (FRT/ICC) and then to the Ethics Committee of Hospital Haroldo Juaçaba (HHJ)/ ICC under the number 4.709.861. All the ethical aspects set out in Resolution No. 466 of 2012 of the National Health Council/Ministry of Health, which sets out the Guidelines and Regulatory Standards for research with human beings under the CONEP (National Research Ethics Commission) standard, were respected.

All the publications from this study involved researchers and members of the medical and multi-professional team at the Haroldo Juaçaba Hospital / Cancer Institute of Ceará.

- Sample calculation 

A case-control study (14) found that low-intensity laser PBMT with a wavelength of 660nm had a greater absence of oral mucositis in head and neck cancer patients treated with radiochemotherapy than the placebo group (59.6% vs. 21.3%). Therefore, we calculated that it was necessary to evaluate 30 patients per study group (chi-square test) in order to obtain a sample that represented the alternative hypothesis of this study with 90% power and 95% confidence, using the Fleiss method. Eight randomized patients per group were analyzed in an interim analysis of one-third of the sample, giving a total sample of 16 patients. Due to significant differences between the two PBMT protocols, the clinical trial was terminated before reaching the total sample (Table 1).

- Participants and clinical scenario: inclusion, exclusion, and withdrawal criteria

Patients over the age of 18 with stages I, II, III and IV oral cancer were selected, following the TNM requirements of the 8th Edition, Staging Manual, of the American Joint Committee on Cancer (AJCC), where T corresponds to Primary Tumor, N to Regional Lymph Nodes and M to Distant Metastases, free of previous antineoplastic treatments, who underwent exclusive treatment with radiotherapy associated with chemotherapy with cisplatin (standard chemotherapy treatment for patients with oral cancer) in combination.

Patients with untreated diabetes mellitus (glycemia > 200 mg/dL or glycated hemoglobin > 7%), using drugs that significantly alter the salivary flow, saliva composition, or taste, using centrally-acting analgesics or anxiolytics and antidepressants, were excluded.

Patients who dropped out of the study (1 patient from each group) or the treatment required a change in the chemotherapy protocol by replacing cisplatin with other chemotherapy drugs, interrupted radiochemotherapy for any reason, developed extreme toxicity, or died were removed from the study.

All patients were treated at the radiotherapy and chemotherapy outpatient clinic of the Haroldo Juaçaba Hospital, a High Complexity Oncology Care Center (CACON), from July 2021 to May 2022.

- Randomization and blinding

The patients were randomly divided into two experimental groups: a gold standard control group and a test group. (Simple) randomization was carried out by a collaborator using the "=random []" command in Microsoft Excel (Microsoft Corporation®) through simple randomization into the two study groups: A and B. After randomization, the randomization numbers were printed on sealed envelopes with the identification of which group they belonged to inside and were opened only by the study's principal investigator at the time of treatment.

The leading researcher had the help of two collaborators, unaware of the group the patients belonged to, to assess oral mucositis and apply the questionnaires, thus making the study blind to the evaluators. In addition, the laser protocol was applied equally in both groups; however, the principal investigator simulated the application of the laser in the test group on days +2 and +4 by switching the device on and off immediately, thus blinding the study to the patients. The evaluator and the supporting statistician were also unaware of the group to which each patient belonged. Thus, only the principal investigator was aware of the groups to which the patients belonged, blinding the patient, the evaluators, and the statistician, making the study triple-blind.

- Study groups and experimental protocol

After signing the Free and Informed Consent Form (Appendix I) and agreeing to take part in the study, clinical and pathological data were collected, along with sociodemographic and dental data (Appendix II) and the evaluation for oral mucositis and visual analog pain scale (Appendix III), in addition to the subjective global assessment produced by the patient (SGAPP) (Appendix I) and the quality of life questionnaire (Appendix II). All of this information were collected at baseline, before the leading researcher applied the first dose of photobiomodulation.

A pre-sealed envelope labeled with each patient's study entry number was opened by the principal investigator, who randomly assigned the patients to one of two study groups: the gold standard control group or the test group. The patients were not given any information and did not know which group they belonged to. Finally, PBMTwas performed, and both groups received the same dose of laser, differing in the application protocol.

The gold standard control group received a daily application of the PBMT protocol (Monday to Friday, i.e., days 1, 2, 3, 4, and 5 of the week). The test group received the protocol three times a week (Monday, Wednesday, and Friday, i.e., days 1, 3, and 5) interspersed with a placebo (Tuesday and Thursday, i.e., days 2 and 4 of the week).

The protocol was carried out using the Therapy XT laser (DMC, São Carlos, SP, Brazil) with 0.1W of power and continuous wavelength light output of 660±10nm (red). The device had a tip with an area of 0.28 mm² (or 0.028 cm²), which, during the applications, was kept in light contact with the treated area.

The dose of preventive PBMT used was the same as that proposed by ANTUNES *et al*. 2013, in which 1J of red light with a wavelength of 660nm was applied continuously to symmetrically distributed points on the labial mucosa, right and left buccal mucosa, right and left lateral borders of the tongue, floor of the mouth and body of the tongue, totaling nine applications per area. All groups received basic oral health prevention protocols (15).

- Clinical-pathological and sociodemographic data collection

The Electronic Patient Record (EPR) was evaluated to collect clinical and pathological data, including age, gender, race, schooling, pTNM, tumor location, presence of nasoenteral tube and/or tracheostomy, radiotherapy modality and doses, and chemotherapy protocol.

- Oral health profile and adequacy of the oral environment

Prior to the first session of radiochemotherapy, the patients had their oral cavity inspected by the principal investigator to assess the soft and hard tissues of the maxillo-mandibular complex. The decayed, missing, and filled teeth (DMFT) index was calculated, and the degree of tooth mobility was assessed (LÖE & SILNESS, 1963). Data was collected in the radiotherapy or chemotherapy department using a clinical dental photophore attached to the head of the main evaluator (Appendix II).

In addition, unstimulated salivary secretion was collected and assessed using the expectoration method. For this method, the subjects remained for three minutes without swallowing, and at the end, they expelled all the saliva stored in their mouths into a graduated container. 3 ml of saline solution was added so that any droplets of saliva adhered to the wall of the container could decant and be divided by the number of minutes the patient had not swallowed (Appendix VI) (16).

- Evaluation of oral mucositis

Every day before each application of PBMT or placebo, the evaluator blinded to the study was responsible for assessing the incidence of oral mucositis using the scores suggested by the World Health Organization. Thus, after training and intra-examiner (kappa = 0.857) and inter-examiner (kappa = 0.921) calibration, mucositis was classified by two researchers as grade 0, when there is no mucositis; grade 1, when there is erythema with no need for intervention; grade 2, when there is an ulcer or pain that does not interfere with food intake; grade 3, when there is severe pain with interference with food intake; grade 4, when there is a risk to life with a need for urgent intervention; and grade 5 when the patient dies (17). 

Patients were also asked about their subjective perception of pain using a Visual Analog Scale (VAS) scale (Appendix V), ranging from 0 to 10, where 0 corresponds to no pain and 10 to the maximum pain ever experienced.

- Body mass index (BMI) and food perception

BMI was calculated on day D0 of the study and on the last day of the radiochemotherapy protocol. The patient was weighed on a conventional scale, and their weight was divided by their height squared to calculate their body mass index (BMI = mass / height²). These data were collected from the ICC's electronic medical records.

During the same periods (before and after the end of radiochemotherapy), the patients answered the Portuguese version of the subjective global assessment produced by the patient (SGAPP). The SGAPP is an inventory developed to assess the nutritional status of cancer patients, previously validated in Brazilian Portuguese. Composed of two blocks, one containing questions for the patient and one containing assessments to be made by a health professional, the scale allows simple summations to obtain a nutritional status score for the evaluated patient. As it is a subjective scale, it must be applied by the same professional to reduce observation bias (18) (Appendix II).

- Quality of life analysis

After applying the SGAPP, the OHIP-14 questionnaire was used to assess the quality of life associated with oral health. This questionnaire was also administered before and at the end of radiochemotherapy. The OHIP-14 is a subjective indicator that measures the disability, discomfort, and handicap attributed to the oral condition through self-assessment and its relationship with quality of life.

It consists of 14 questions and is a reduced version of the OHIP-49. It is also numbered on a Lickert-type scale, with answers ranging from (1) never, 2 (rarely), 3 (sometimes), 4 (repeatedly), and 5 (always) (Appendix II). Validated since the 1990s and widely used in dental research, it is divided into seven domains: (D1) Functional limitation (questions 1 and 2), (D2) Physical pain (questions 3 and 4), (D3) Psychological discomfort (questions 5 and 6), (D4) Physical limitation (questions 7 and 8), (D5) Psychological limitation (questions 9 and 10), (D6) Social limitation (questions 11 and 12) and (D7) Disability (questions 13 and 14). The seven domains constitute the overall quality of life scale (ranging from 14 to 70).

- Statistical analysis

The data was expressed as absolute and percentage frequencies and compared using Fisher's exact test, Pearson's chi-square test or mean and standard deviation, submitted to the Shapiro-Wilk normality test and compared using the Mann-Whitney test (non-parametric data). The analyses were conducted using SPSS v20.0 software for Windows, with a 95% confidence level.

## Results

- Characterization of the sample

Most of the patients assessed were male, with a mean age of 64.50±10.42 to 68.50±15.33 years, brown, and with a low level of education. None of these variables showed any significant difference between the experimental groups. The group treated with continuous days of PBMT had a higher frequency of T4 tumors than those treated with PBMT on alternate days (*p*=0.029). However, in both groups, most of the patients had N0 nodal status (*p*=0,494), without distant metastases (*p*=1.000), with tumors equally distributed in the tongue, followed by lip and palate (*p*=1.000) and without the need for probing (*p*=0.590) or tracheostomy (*p*=0.248) (Table 1).

From a therapeutic point of view, the most frequent RT modality was intensity-modulated radiotherapy (*p*=0.522), with no need for interruptions (*p*=0.590). There was no difference in the total dose of radiotherapy between the two groups (*p*=0.632), nor the number of fractions (*p*=0.365) or radiotherapy time (*p*=0.753). Most of the patients in both groups used cisplatin chemotherapy concomitantly with radiotherapy (*p*=0.133), and most patients did not require a dose reduction (*p*=0.590) (Table 1).

Odontologically, most patients did not have periodontal disease (*p*=0.131), and the number of decayed, missing, and filled teeth was high in both groups (*p*=0.891) (Table 1).

**Table 1:**
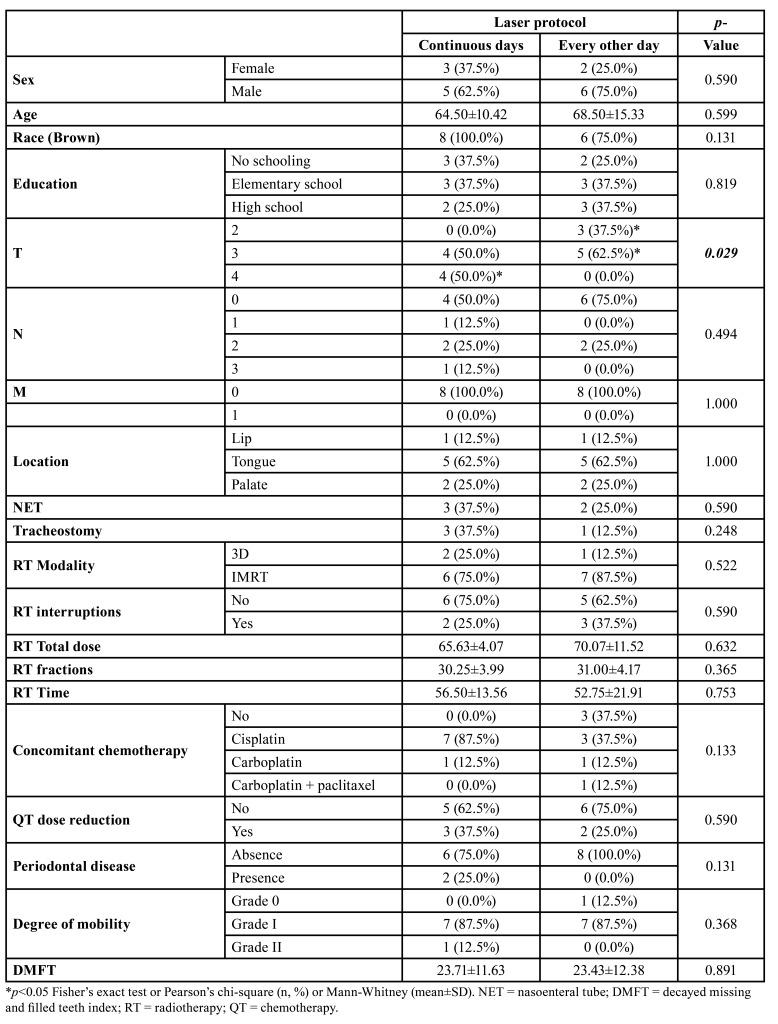
Clinical-pathological profile of patients with head and neck tumors undergoing radiotherapy and submitted to preventive photobiomodulation protocols with application on continuous and alternate days.

- Analysis of mucositis

Most of the patients had some form of oral mucositis during head and neck radiotherapy. There was no difference between the groups in the frequency of maximum oral mucositis scores (*p*=0.198) or scores greater than or equal to 2 (*p*=0.131). When the events were assessed individually, a significant increase in the frequency of grade 2 and grade 3 oral mucositis was observed in the group treated with PBMT every other day compared to continuous days (*p*<0.001). The relative risk of grade II or higher oral mucositis was 6.79 (95%CI = 2.65-17.44) times higher in the every-other-day PBMT group (Table 2).

There was no significant difference in the maximum oral pain score experienced by the two groups throughout the PBMT protocols (*p*=0.915), but evaluating the events individually, the group treated with PBMT on alternate days compared to continuous days had higher average pain scores (*p*<0.001) (Table 2).

**Table 2:**
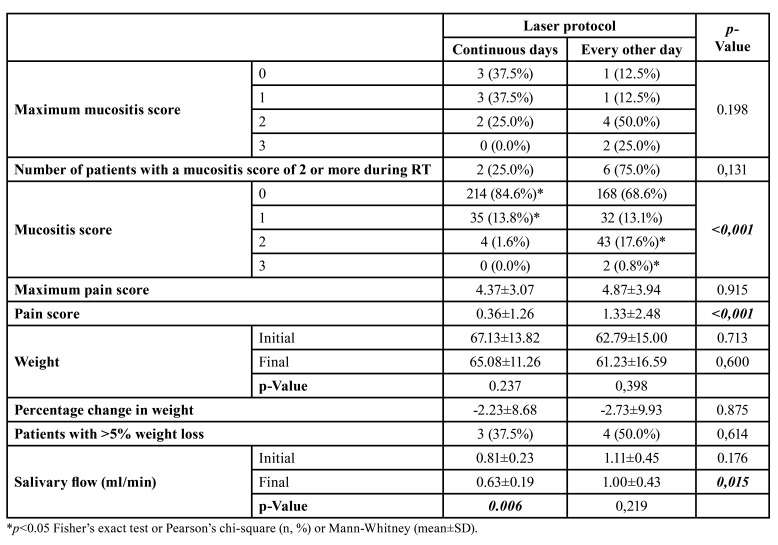
Profile of oral mucositis, oral pain, and weight loss in patients with head and neck tumors undergoing radiotherapy and subjected to preventive photobiomodulation protocols with application on continuous and alternate days.

On days D11, D12, D31 and D32, the group that received preventive laser therapy on alternate days showed significantly higher pain scores (*p*=0.027; *p*=0.027; *p*=0.046; *p*=0.046) respectively (Table 3).

Evaluating day by day, patients who underwent PBMT protocols every other day showed higher oral mucositis scores on D14 (*p*=0.047), D15 (*p*=0.047), D16 (*p*=0.047), D17 (*p*=0.047), D18 (*p*=0.047), D19 (*p*=0.047) and D20 (*p*=0.047) and pain on D12 (*p*=0.027), D31 (*p*=0.046) and D32 (*p*=0.036) (Supplement 1).

There was no significant difference in weight variation between the two groups (*p*>0.05). However, the group treated with PBMT on continuous days showed a greater reduction in salivary flow at the end of radiotherapy than the protocol on alternate days (*p*=0.006) (Table 2). The profile of food intake and oral health-related quality of life did not differ significantly between the groups (Table 4).

**Table 3:**
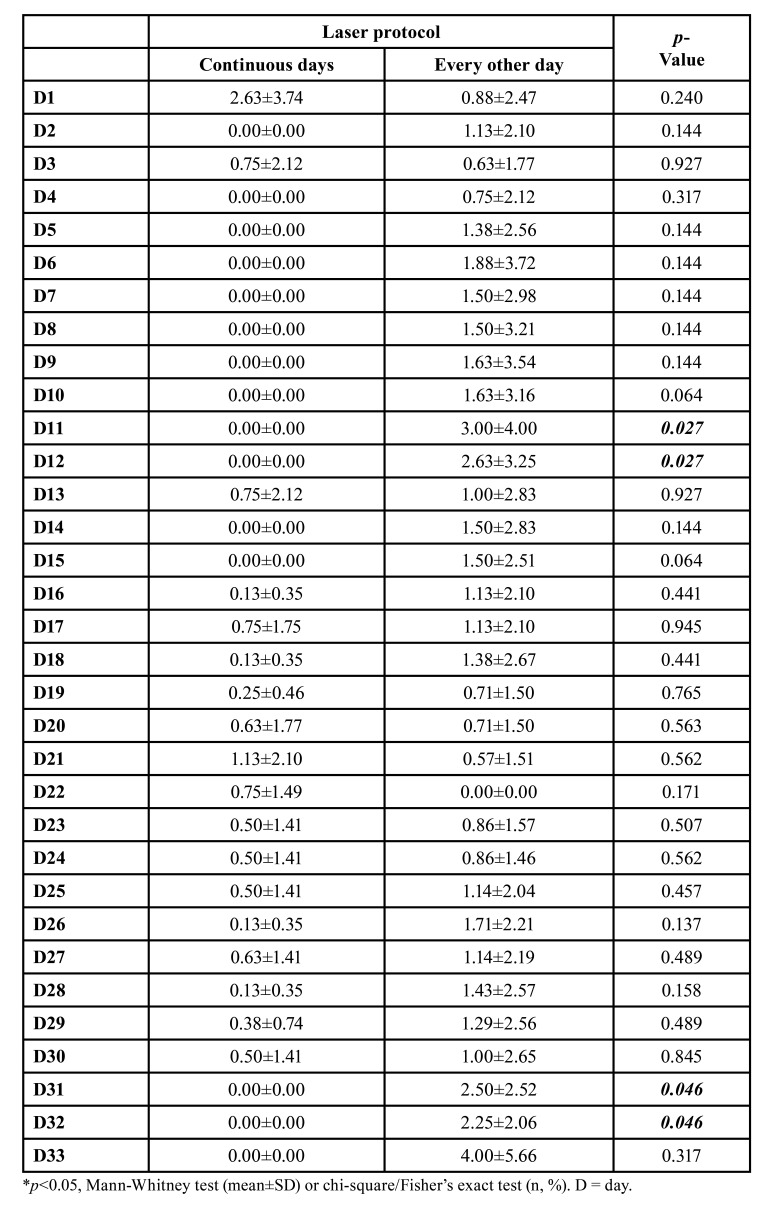
Mean±SD of pain scores of patients with head and neck tumors undergoing radiotherapy and submitted to preventive photobiomodulation protocols with application on continuous and alternate days.

**Table 4:**
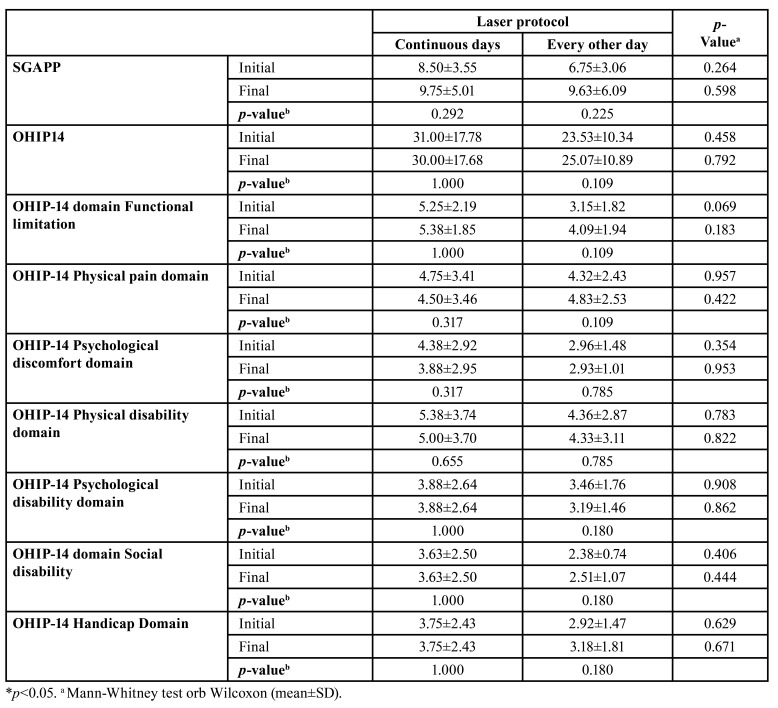
Profile of dietary intake and oral health-related quality of life of patients with head and neck tumors undergoing radiotherapy and subjected to preventive photobiomodulation protocols with application on continuous and alternate days.

## Discussion

OM is one of the most severe adverse effects during oncological treatment, especially in patients receiving radiotherapy in the head and neck region, affecting around 40% to 90% of these individuals (19). Even with advances in radiotherapy modalities, such as Intensity Modulated RT (IMRT) or Volumetric Arc Therapy (VMAT), which allow for better preservation of organs at risk, especially the oral mucosa, the prevalence of MO is still considerably high, especially in patients with tumors in the mouth (20).

The development of OM during treatment can lead to systemic consequences, such as a decline in nutritional status due to reduced oral intake, resulting in malnutrition and even cachexia (4,5). These problems can interrupt treatment, leading to a worse prognosis and a drop in overall survival (21).

Among the treatments recommended for this condition, PBMT has proven effective and is the first choice for treating and preventing OM (12). There is a constant search in the literature for preventive protocols with the greatest efficacy, so different protocols have been studied and approached, but there are still no standard protocols for preventing this adverse effect (12,13).

Our study aimed to evaluate OM prevention protocols based on their efficacy, cost-effectiveness, and clinical applicability in an oncological reference center for patients undergoing radiotherapy for oral cancer, comparing daily therapy with therapy every other day.

The majority of the assessed patients were male, brown, with low levels of education, with a mean age of 64.50±10.42 to 68.50±15.33 years. These findings are in accordance with those in the literature, which found a higher prevalence of oral and oropharyngeal cancer in brown males aged 65 or over and with incomplete primary education, reinforcing the influence of these factors on the epidemiology of cancer (22).

Although these factors are sources of critical epidemiological data, none of these variables showed any significant difference between the experimental groups. The group treated with continuous days of PBMT had a higher frequency of T4 tumors than the patients treated every other day, but in both groups, most of the patients had N0 nodal status with no distant metastases. This is an important finding, given that some studies show that patients classified as T3-T4 tend to have a higher prevalence of distant metastases, mainly when associated with positive lymph nodes (22,23).

However, the tumors were equally distributed, mostly in the tongue, followed by the lip and palate. This is in line with other studies in which tumors in the oral cavity were more prevalent in the tongue and palate, followed by the retromolar region, the floor of the mouth, and the lip. The jugal mucosa and gingiva are also commonly affected (22).

Most patients in both groups used chemotherapy with cisplatin concomitant with radiotherapy, and most patients did not require a dose reduction. These data corroborate those in the literature, where chemotherapy based on platinum coordination complexes (cis-DDP) is described as the main line of chemotherapy in the treatment of squamous cell carcinomas in the head and neck, esophagus, endometrium, gastric and lung regions, acting through DNA alkylation and cytotoxicity (24).

Most patients did not have periodontal disease, and both groups had a high number of decayed, missing, and filled teeth. This result reinforces the profile of patients diagnosed with head and neck cancer, where we see patients with poor oral health, leading to high DMFT indexes (25).

There was no significant difference in weight variation between the two groups, although the literature reports an association between the presence of OM and its influence on functions such as voice quality, swallowing, lip and tongue health, and salivary flow (26). However, it was observed that the group treated with PBMT on continuous days showed a greater reduction in salivary flow at the end of radiotherapy compared to the protocol on alternate days. Radiotherapy and chemotherapy protocols reduce salivary flow (25,26).

The profile of food intake and quality of life-related to oral health did not differ significantly between the groups. However, the literature reinforces the association between tooth loss and its significant effects on oral health, which affect chewing ability, leading to restricted consumption of various foods and hindering phonation and aesthetics, impacting the patient's quality of life (27).

From a therapeutic point of view, the most frequent modality was intensity-modulated radiotherapy (IMRT), with no difference in total dose, number of fractions, or radiotherapy time between the two groups. IMRT has been frequently used as a therapeutic line in oral cancers, especially in more advanced cases of T3 and T4 staging, because it has an approach that optimizes and maximizes irradiation to the tumor volume. This radiotherapy modality delivers non-uniform radiation beam intensities, thus minimizing damage to adjacent tissues and helping to reduce adverse effects, including MO (28).

Even though IMRT is a healthy tissue-sparing modality, most patients had MO at some point during the RT treatment. There was no difference between the groups in the frequency of MO scores greater than or equal to 2. However, when the events were assessed individually, a significant increase in the frequency of grade 2 and grade 3 MO was observed in the group treated with PBMT every other day compared to continuous days.

We then observed that the relative risk of oral mucositis grade II or higher was 6.79 (95%CI = 2.65-17.44) times higher in the PBMT every other day group. Similarly, we observed that the group treated with PBMT every other day had higher average pain scores than the continuous group.

The literature shows that using PBMT continuously contributes to a better clinical benefit from the low-power laser, both in terms of reducing OM and pain scores (13). Our study aimed to find an effective, cost-effective protocol that would optimize the clinical routine of dental care in an oncology center, showing a similar benefit on alternate days of PBMT to its use on continuous days, but we observed a different set of results.

These findings are justified by the daily cell death-inducing effect of RT, in which, as well as inducing cell apoptosis through direct DNA damage, it also triggers a series of biological events in tissue cells, leading to oxidative stress and the release of reactive oxygen species, which activate the immune response, producing cytokines that have repercussions on tissue damage (29,8).

On the other hand, the low-power laser induces anti-inflammatory, analgesic, and healing effects, which, through its direct action on cell metabolism, optimizes the immediate influx of oxygen and the resumption of the respiratory chain, accelerating the synthesis of intracellular adenosine triphosphate (ATP), it ends up contributing to tissue regeneration (11). As a result, daily applications of PBMT during daily RT treatment may result in a better clinical benefit from the low-power laser.

Although this is a non-superiority study aimed at finding clinical protocols that contribute to daily dental care in cancer centers, we were able to observe the great importance of the presence of qualified professionals to support patients during RT, especially in patients with HNC who receive high doses of radiation in the oral cavity region. Even using RT modalities that spare healthy tissue, most patients develop MO at some point, and in order to prevent and treat this adverse effect with greater scientific evidence, it is necessary to use PBMT, which is more clinically effective daily.

Despite a limited sample, we were able to carry out a well-designed study showing that the low-power laser reduces the severity and pain of OM and is most clinically effective with daily applications. However, we suggest that further clinical trials are carried out to validate this continuous PBMT approach.
